# Lanthanum Carbonate Reduces Urine Phosphorus Excretion: Evidence of High-Capacity Phosphate Binding

**DOI:** 10.3109/0886022X.2011.649657

**Published:** 2012-01-17

**Authors:** Michael Pennick, Lynne Poole, Kerry Dennis, Michael Smyth

**Affiliations:** 1 Shire Pharmaceuticals Group Plc, Basingstoke, UK; 2 Modulus Medical, Berkshire, UK

**Keywords:** lanthanum carbonate, sevelamer hydrochloride, chronic kidney disease, phosphate binder, hyperphosphatemia

## Abstract

The effectiveness of phosphate binders can be assessed by evaluating urinary phosphorus excretion in healthy volunteers, which indicates the ability of the phosphate binder to reduce gastrointestinal phosphate absorption. Healthy volunteers were enrolled into one of five separate randomized trials; four were open label and one double blind. Following a screening period of <28 days, participants received differing tablets containing lanthanum carbonate [LC, 3000 mg/day of elemental lanthanum (in one study other doses were also used)]. Participants received a standardized phosphate diet and remained in the relevant study center throughout the duration of each treatment period. The end point in all studies was the reduction in urinary phosphorus excretion. Reductions in mean 24-h urinary phosphorus excretion in volunteers receiving a lanthanum dose of 3000 mg/day were between 236 and 468 mg/day over the five separate studies. These data in healthy volunteers can be used to estimate the amount of reduction of dietary phosphate absorption by LC. The reduction in 24-h urinary phosphorus excretion per tablet was compared with published data on other phosphate binders. Although there are limitations, evidence suggests that LC is a very effective phosphate binder in terms of binding per tablet.

## INTRODUCTION

Phosphate binders are usually required to restore phosphate balance in patients with advanced chronic kidney disease (CKD). The effectiveness of such binders depends on their ability to bind dietary phosphate in the gut and the stability of the resulting insoluble complex in the presence of normal contents of the gastrointestinal tract, thus preventing absorption. Several phosphate binders are currently available, including calcium-based agents (calcium carbonate and calcium acetate). However, calcium-based agents may increase calcium load and the risk of vascular calcification.^1^–^3^

Lanthanum carbonate [(LC) FOSRENOL, Shire Pharmaceuticals, Basingstoke, UK], sevelamer hydrochloride [(SH) Renagel, Genzyme Therapeutics, Cambridge, MA, USA], and sevelamer carbonate (Renvela, Genzyme Therapeutics, Cambridge, MA, USA) are non-calcium-containing phosphate binders licensed for use in CKD patients.^4^–^6^ In vitro data indicate that LC has a higher binding affinity for phosphate than SH and LC's phosphate binding is not affected by acidity or the presence of bile acids.^7^

In healthy people and patients with the early stages of CKD, serum phosphorus levels are maintained within the normal range by complex homeostatic mechanisms with the kidney as one of the main regulatory organs for maintenance of phosphate balance.^8^ As such individuals are in neutral phosphate balance, measurement of urinary phosphorus excretion can be used as an estimate of intestinal phosphate absorption.^5^

This article presents results from five healthy volunteer studies examining the ability of LC to bind dietary phosphate.

## MATERIALS AND METHODS

### Trial Designs

Each randomized trial of healthy volunteers was a single center study; studies 1, 2, and 3 were in UK centers; study 4 was in a center in the United States; and study 5 was in a center in Japan. All were carried out in accordance with the principles of the 18th World Medical Assembly (Helsinki 1964) and amendments, and local ethical and legal requirements. Informed consent was obtained from all volunteers. In all studies (abbreviated details in Table 1), volunteers were randomized following a screening period of up to 28 days and participants remained in the study centers for the study period only and not during washout periods. Restrictions on concomitant medication use occurred in each study. The only permitted medications were hormonal contraceptives and treatments required for adverse events. Vitamin supplements were excluded during the studies. Study 5 was a double-blind study and studies 1–4 were all open label.

**Table 1:** Summary of studies of lanthanum carbonate (LC).

Study	Description	*n*	Phosphorus intake (mg/day)	Treatments (doses)	Details
Study 1	Parallel-group, dose-ranging study	70	∼2000	LC^a^	• Taken with 200 mL water
				• 750 mg/day	• Three times daily for 6 days, with one dose given on the morning of day 7
				• 1500 mg/day	• Average 24-h urinary phosphorus excretion was calculated for the 3 days before dosing (baseline) and the final 3 days of treatment
				• 3000 mg/day	
				• 4500 mg/day	
				• 6000 mg/day Control	
Study 2	Three-way crossover, pharmacodynamic equivalence study	56	Standard	LC	• Taken with 400 mL water
				• 3000 mg/day^a^	• Three times daily for 3 days, with one dose given on the morning of day 4
				• 4 × 250 mg	• At least 14 days washout was allowed between treatment periods
				•2 × 500 mg	• Average 24-h urinary phosphorus excretion was calculated for the 2 days before dosing (baseline) and the 3-day treatment periods
				• 1 × 1000 mg	
				All three times daily	
Study 3	Two-way crossover, alternative formulation study	52	∼1300	LC	• Taken with 200 mL water
				• 3000 mg/day^a^	• Three times daily for 3 days, with one dose given on the morning of day 4
				• 4 × 250 mg	
				• 1 × 1000 mg formulation	• At least 14 days washout between treatment periods
				All three times daily	• Average 24-h urinary phosphorus excretion was calculated for the day before dosing (baseline) and the 3-day treatment periods
Study 4	Two-way crossover, optimized formulation study	57	∼1300	LC	• Taken with 240 mL water
				• 3000 mg/day^a^ (4 × 250 mg three times daily)	• Three times daily for 3 days, with one dose given on the morning of day 4
				• Optimized formulation (batch A)	• At least 10 days washout between treatment periods
				• Alternative formulation (batch B)	• Average 24-h urinary phosphorus excretion was calculated for the 2 days before dosing (baseline) and the 3-day treatment periods
Study 5	Parallel-group, placebo-controlled study	9	∼1200	LC	• Taken with 150 mL water
				• 3000 mg/day^a^	• Three times daily for 5 days
				• 4 × 250 mg, three times daily	• 24-h urinary phosphorus excretion was calculated on the day before dosing (baseline) and compared with that on the final day of treatment
				• Matching placebo	

Note:

a Elemental lanthanum.

#### Study 1

This parallel-group study involved 70 volunteers randomized to receive one of six treatments. LC at five different daily doses—1 × 250 mg tablet, 1 × 500 mg tablet, 1 × 1000 mg tablet, 2 × 750 mg tablets, and 2 × 1000 mg tablets—with 200 mL of water three times daily (elemental lanthanum: 750, 1500, 3000, 4500, 6000 mg) was compared with control. Participants entered the unit 8 days before first dosing and received their LC tablet(s) with 200 mL water three times daily for 6 days with one dose given on the morning of day 7. Study participants received a standardized phosphate diet throughout the study period (approximately 2000 mg of elemental phosphorus per day). The primary objective was to investigate the relationship between LC dose and urinary phosphorus excretion. The highest dose of 6000 mg was used to assess the potential maximum dose for clinical use.

#### Study 2

This study was designed to establish pharmacodynamic equivalence of equal doses of three different tablet strengths of LC. Reducing the number of tablets a patient requires each day may improve patient compliance. Under the three-way crossover study design, 56 volunteers were randomized into six treatment sequences, with at least nine volunteers per sequence, immediately before the first dose of the study. Individuals received 1000 mg of elemental lanthanum either as 4 × 250 mg LC tablets or 2 × 500 mg tablets or 1 × 1000 mg tablet with 400 mL of water three times daily for 3 days and one dose in the morning of day 4. There were three treatment periods of 4 days' dosing with at least 14 days washout period between the last dose of each treatment period and the first dose of the subsequent treatment period. A standard menu was followed to ensure the same daily phosphate intake for each treatment period.

#### Study 3

In this crossover study, 52 volunteers were randomized to two treatment sequences to receive 1000 mg of elemental lanthanum, in one of two alternative formulations, either as the (at the time) existing formulation of 4 × 250 mg LC tablets or a new formulation of 1 × 1000 mg tablet with 200 mL of water three times daily for 3 days and one dose in the morning of day 4. Following a washout period of at least 14 days, participants crossed over to the alternative LC formulation for 4 days' dosing. Study volunteers received a standardized phosphate diet throughout the study period (approximately 1300 mg of elemental phosphorus per day).

#### Study 4

Fifty-seven volunteers were randomized to one of two treatment sequences to receive tablets with two different dissolution characteristics (batch A: optimized formulation or batch B: alternative formulation) in a crossover study. Tablets in batch A passed a 10 dips/min dissolution test, whereas tablets in batch B failed 10 and 20 dips/min dissolution tests and passed a 30 dips/min dissolution test. Participants received 1000 mg of elemental lanthanum as 4 × 250 mg of LC tablets with 240 mL water three times daily for 3 days, with 4 × 250 mg dose given in the morning of day 4. After a washout period of at least 10 days between dosing periods, volunteers crossed over to the other batch. During study periods, individuals received a standardized phosphate diet (approximately 1300 mg of elemental phosphorus per day).

#### Study 5

This placebo-controlled, parallel-group study involved nine Japanese healthy volunteers randomized into one of two groups. Participants received either 1000 mg of elemental lanthanum as 4 × 250 mg LC tablets with 150 mL water three times daily or matching placebo for 5 days. During the study periods individuals received a standard phosphate diet (approximately 1200 mg of elemental phosphorus per day).

### Diets

Participants consumed food from phosphate-standardized menus while resident in study centers. LC was taken during or immediately after consumption of the standard meals. No other phosphate-containing foods or drinks were permitted during treatment periods.

### Urine Collection

All urine samples were collected for 24-h periods starting and ending before the morning dose of LC. During collection, urine was stored at 4°C. The total urine volume over the collection period was recorded. Aliquots were transferred into polypropylene containers and stored frozen (−20°C) before analysis of phosphorus concentration by colorimetric methods.

### Assessments

In each study, 24-h urinary phosphorus excretion was measured in the pharmacodynamic population using methods consistent with those used previously to determine the phosphate-binding capacity of SH.^5^ Any participant who vomited during the days that measurement of 24-h urinary phosphorus excretion was being made was excluded from studies 1–4.

In study 1, baseline assessments were made in the 3 days before dosing and treatment was given from days 8 to 13 and on the morning of day 14. In this study, 24-h urinary phosphorus excretion was measured in the final 3 days of treatment. Although the dosing period was longer in this study, the end point was still the average daily urinary phosphorus excretion over 3 days of the treatment period. In study 2, 24-h urinary phosphorus excretion was measured the day before first dosing, on each dosing day, and for 48 h after dosing. The end point for assessment of pharmacodynamics in study 2 was the average daily reduction in urinary phosphorus excretion during the 3-day LC treatment period. In study 3, 24-h urinary phosphorus excretion was measured for 2 days before dosing and the 3 days of full dosing. The end point was the average level of daily urinary phosphorus excretion during the 3-day LC treatment period. In study 4, measurement of 24-h urinary phosphorus excretion was made for 48 h before the first dose and then on each day of dosing. The end point was the average daily urinary phosphorus excretion over the 3-day dosing period. In study 5, 24-h urinary phosphorus excretion was measured the day before dosing, on each of the 5 dosing days, and for 48 h after the final day of dosing. The reduction in 24-h urinary phosphorus excretion during the final day of dosing was compared with baseline. In summary, studies 1–4 all had the reduction in 24-h urinary phosphorus excretion averaged over 3 days as the end point. Only in study 5 was the end point reduction in urinary phosphorus excretion based on just the value of 1 day.

Adverse events, physical examination, vital signs, and clinical laboratory tests were assessed during screening and throughout each study. Serum phosphorus and calcium levels were also assessed during the studies.

### Statistical Methods

The safety population was defined as all participants who were randomized and received at least one dose of study medication in all studies. The pharmacodynamic population in studies 1, 3, and 4 included all individuals in the safety population who completed all urine collections in all treatment groups and consumed at least 95% of food in all treatment periods. The pharmacodynamic population in studies 2 and 5 included all individuals in the safety population who completed all urine collections in all treatment groups.

The end points of studies 2, 3, and 4 were analyzed using a mixed effects linear model with sequence, treatment (or batch for study 4), and period as fixed effects, and subject within sequence as a random effect. In studies 2 and 3, baseline was also included as a covariate. An analysis of variance with treatment as a factor was used in study 1. In study 5, the reductions in 24-h urinary phosphorus within each treatment group were analyzed using a paired *t*-test.

## RESULTS

Baseline characteristics of volunteers in the safety population of treatment groups in study 1 are listed in Table 2. The baseline characteristics of volunteers in the safety population of all five studies are listed in Table 3.

**Table 2:** Baseline characteristics for study 1 at total doses of 750, 1500, 3000, 4500, and 6000 mg lanthanum per day and control.

Parameter	750 mg/day	1500 mg/day	3000 mg/day	4500 mg/day	6000 mg/day	Control
	
*n*	10	10	10	10	10	10
Age, mean ± SD (range) (years)	26.1 ± 5.7 (20–38)	27.2 ± 7.4 (18–42)	25.4 ± 5.2 (20–37)	23.9 ±3.0 (20–28)	23.4 ±3.4 (20–31)	25.9 ±3.8 (19–31)
Gender						
Male	6 (60.0)	7 (70.0)	6 (60.0)	1 (10.0)	2 (20.0)	4 (40.0)
Female	4 (40.0)	3 (30.0)	4 (40.0)	9 (90.0)	8 (80.0)	6 (60.0)
Race						
Caucasian	9 (90.0)	9 (90.0)	9 (90.0)	9 (90.0)	8 (80.0)	10 (100.0)
Black	0(0)	0(0)	0(0)	1 (10.0)	0(0)	0(0)
Asian	1 (10.0)	1 (10.0)	1 (10.0)	0(0)	1 (10.0)	0(0)
Other	0(0)	0(0)	0(0)	0(0)	1 (10.0)	0(0)
Weight, mean ± SD (range) (kg)	73.5 ±8.3 (60–83)	70.8 ± 13.9 (51–93)	72.3 ± 12.6 (54–91)	69.8 ± 10.7 (56–85)	63.8 ±9.6 (54–81)	68.9 ±6.5 (56–76)
Serum phosphorus, mean ± SD (range) (mg/dL)	4.1 ±0.3 (3.7–5.0)	3.9 ±0.4 (3.4–4.3)	4.1 ± 0.4 (3.7–4.6)	4.1 ± 0.5 (3.7–5.0)	4.1 ±0.5 (3.4–5.0)	3.7 ±0.4 (3.4–4.6)
Serum calcium, mean ± SD (range) (mg/dL)	9.8 ±0.4 (9.2–10.4)	9.8 ±0.2 (9.6–10.0)	9.9 ± 0.2 (9.6–10.4)	9.7 ± 0.3 (9.2–10.0)	9.7 ±0.2 (9.6–10.0)	9.9 ±0.2 (9.6–10.0)

Notes: Data are presented as *n* (%), unless otherwise stated. Data relate to the safety population (with the exception of serum phosphorus and calcium, which are from the pharmacodynamic population) in all study groups.

**Table 3:** Baseline characteristics with lanthanum carbonate (LC) at a dose of 3000 mg/day of elemental lanthanum.

	Study				
	
Parameter	Study 1	Study 2	Study 3^a^	Study 4	Study 5
	
*n*	10	56	52	57	6
Age, mean ± SD (range) (years)	25.4 ± 5.2 (20–37)	25.2 ± 5.0 (18–40)	23.3 ± 3.2 (18–31)	29.2 ± 10.8 (18–55)	23.3 ± 1.8 (21–26)
Gender					
Male	6 (60.0)	27 (48.2)	25 (48.1)	37 (64.9)	6 (100)
Female	4 (40.0)	29 (51.8)	27 (51.9)	20 (35.1)	–
Race					
Caucasian	9 (90.0)	54 (96.4)	46 (88.5)	12 (21.1)	–
Black	0(0)	0(0)	3 (5.8)	9 (15.8)	–
Asian	1 (10.0)	1 (1.8)	1 (1.9)	8 (14.0)	6 (100)
Other	0(0)	1 (1.8)	2 (3.8)	28 (49.1)	–
Weight, mean ± SD (range) (kg)	72.3 ± 12.6 (54–92)	68.4 ± 10.8 (50–95)	67.2 ± 9.0 (47–95)	72.6 ± 12.4 (47.2–106.8)	60.2 ± 6.5 (54–72)
Serum creatinine, mean ± SD (range) (mg/dL)	Not recorded	0.91 ±0.11 (0.6–0.9)	A: 0.82 ±0.11 (0.7–1.0)	0.95 ±0.23 (0.5–1.8)	1.03 ± 0.08 (1.0–1.2)
			B: 0.84 ±0.15 (0.6–1.1)		
Serum phosphorus, mean ± SD (range) (mg/dL)	4.1 ±0.4 (3.7–4.6)	3.6 ±0.5 (2.5–4.6)	A: 3.1 ±0.4 (2.2–4.0)	3.6 ±0.5 (2.5–5.0)	3.7 ±0.3 (3.3–4.2)
			B: 3.1 ±0.5 (1.5–4.0)		
Serum calcium, mean ± SD (range) (mg/dL)	9.9 ±0.2 (9.6–10.4)	9.9 ±0.4 (9.2–10.8)	A: 10.1 ±0.3 (9.6–10.8)	9.7 ±0.3 (8.6–10.4)	9.1 ±0.3 (8.7–9.3)
			B: 10.0 ± 0.4 (9.2–10.8)		

Notes: Data are presented as *n* (%), unless otherwise stated. Data relate to the safety population in all studies with the exception of phosphorus and calcium data for study 1; studies 2, 3, and 4 were crossover studies; study 1 only included patients receiving a daily dose of 3000 mg LC; and study 5 only included patients receiving LC.

a Patients in arm A received 4 × 250 mg LC tablets first, those in arm B received 1 × 1000 mg LC tablet first.

The number of volunteers included in the pharmacodynamic population in the three crossover studies was 47, 48, and 33 in studies 2, 3, and 4, respectively. In study 1, nine volunteers were included in the pharmacodynamic population receiving a daily dose of 3000 mg LC, whereas in study 5, six volunteers were included in the pharmacodynamic population receiving LC.

### 24-h Urinary Phosphorus Excretion

**Figure 1:**
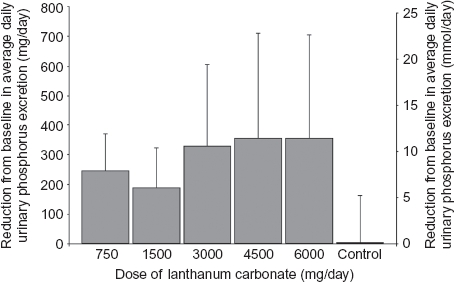
Mean ± SD reduction from baseline in average daily urinary phosphorus excretion at different doses of lanthanum carbonate (study 1).

All five LC groups in study 1 showed a decrease from baseline in urinary phosphorus excretion values, with the highest dose groups (3000–6000 mg/day) showing the greatest change (Figure 1).

Reductions in mean 24-h urinary phosphorus excretion from baseline in the pharmacodynamic population with a LC dose of 3000 mg/day across all five studies are shown in Table 4. This LC dose reduced urinary phosphorus levels by between 236 and 468 mg/day (7.6–15.1 mmol/day). In those studies where they were directly compared, the different LC formulations were shown to be pharmacodynamically equivalent. As expected, homeostasis was maintained by the normal kidney function in healthy volunteers; therefore, LC treatment was not associated with clinically significant changes in serum phosphorus or calcium levels in any of the studies.

**Table 4:** Reduction in 24-h urinary phosphorus excretion from baseline with lanthanum carbonate (LC) at a dose of 3000 mg/day of elemental lanthanum.

	Urinary phosphorus excretion, mg/day (mmol/day)		
	
Study and formulation (all given three times daily)	At baseline	At end point	Reduction from baseline
Study 1 (*n* = 9)			
1 × 1000 mg tablet	992 ±253 (32.0 ±8.2)	662±154 (21.4±5.0)	329 ±276 (10.6 ±8.9)
Study 2 (*n* = 47)			
4 × 250 mg tablets	592 ±233 (19.1 ±7.5)	353±133 (11.4±4.3)	239 ±183 (7.7 ±5.9)
2 × 500 mg tablets	663 ±252 (21.4±8.1)	370±164 (11.9 ± 5.3)	294±177 (9.5 ±5.7)
1 × 1000 mg tablet	674±310 (21.8 ± 10.0)	356±144 (11.5 ± 4.6)	318±294 (10.3 ±9.5)
Study 3 (*n* = 48)			
4 × 250 mg tablets	637±195 (20.6 ±6.3)	393±130 (12.7 ±4.2)	244±164 (7.9 ±5.3)
1 × 1000 mg tablet	646 ±167 (20.8 ±5.4)	410±142 (13.2 ±4.6)	236 ±167 (7.6 ±5.4)
Study 4 (*n* = 33)			
Batch A (4 × 250 mg tablets)	952±155 (30.7 ±5.0)	643 ±116 (20.7 ±3.7)	309 ±149 (10.0 ±4.8)
Batch B (4 × 250 mg tablets)	921 ±177 (29.7 ±5.7)	641 ±143 (20.7 ±4.6)	279 ±168 (9.0 ±5.4)
Study 5 (*n* = 6)			
4 × 250 mg tablets	680±211 (21.9 ±6.8)	212±99 (6.8±3.2)	468±167 (15.1 ±5.4)

Notes: Data relate to the pharmacodynamic populations; studies 2, 3, and 4 were crossover studies; study 1 only included patients receiving a daily dose of 3000 mg LC; and study 5 only included patients receiving LC. In study 4, batch A: optimized formulation; batch B: alternative formulation. Data are mean ± SD.

### Tolerability

Overall, LC was well tolerated and there were no serious adverse events in any of these studies. There was no evidence of any dose-related increase in adverse events across the LC groups in study 1. Most adverse events across the LC groups in study 1 were mild in intensity and the most common were headache, nausea, and abdominal pain. With regard to treatment-related adverse events, gastrointestinal disorders (nausea or vomiting) were experienced by two (20%) participants and headache was experienced by one individual (10%) in the group receiving an elemental LC dose of 3000 mg/day in study 1. In study 2, the most common adverse event was nausea, which was experienced by six (12%) volunteers receiving the reference (4 × 250 mg tablets) formulation, 10 (20%) volunteers receiving the existing (2 × 500 mg tablets) formulation, and 10 (19%) volunteers receiving the optimal (1 × 1000 mg tablets) formulation. In study 3, headache was experienced by two (4%) individuals receiving 4 × 250 mg tablet dosing and six (12%) individuals receiving 1000 mg tablet dosing. Nausea was experienced by two (4%) participants receiving 250 mg tablets and by three (6%) participants receiving 1000 mg tablets. One (2%) participant receiving 250 mg tablets and three (6%) participants receiving 1000 mg tablets experienced abdominal pain. In study 4, the most frequently reported adverse events were headache [two (4%) participants receiving batch A and five (10%) participants receiving batch B] and dizziness [three (6%) participants receiving batch A and two (4%) participants receiving batch B]. Dyspepsia was experienced by three (6%) volunteers receiving batch A but no one receiving batch B and pharyngolaryngeal pain was experienced by no participants receiving batch A but three (6%) participants receiving batch B. There were no adverse events in study 5.

## DISCUSSION

Control of phosphate overload in patients with CKD is now recognized as an important target for reducing the high mortality rate associated with this condition^9^,^10^ and current practice guidelines recommend aggressive treatment of hyperphosphatemia to achieve lower serum phosphorus targets.^11^

The Kidney Disease Outcomes Quality Initiative nutritional guidelines recommend maintenance of protein intake at 1.2 g/kg body weight per day for hemodialysis patients.^12^ With an average phosphate content of 10–12 mg/g protein, a 70 kg patient should therefore consume 840–1008 mg phosphate (27.1–32.5 mmol elemental phosphorus) per day.^11^ Based on the conservative estimate that 60% of ingested phosphate is absorbed from the gastrointestinal tract,^13^ a typical hemodialysis patient will absorb up to 4200 mg (135.5 mmol) phosphate per week. As thrice-weekly hemodialysis will only remove approximately 2400 mg (77.4 mmol) phosphate,^11^ absorption of approximately 1800 mg (58.1 mmol) phosphate per week must be prevented to achieve neutral phosphate balance.

Recommended protein intake is slightly higher for peritoneal dialysis patients (1.2–1.3 g/kg bodyweight per day)^11^ compared with their counterparts on hemodialysis. A 70 kg peritoneal dialysis patient may therefore consume up to 1092 mg phosphate (35.3 mmol elemental phosphorus) per day, resulting in absorption of 4586 mg (148.1 mmol) phosphate per week (assuming 60% absorption^13^). As peritoneal dialysis removes only about 315 mg (10.2 mmol) phosphate per day,^11^ absorption of approximately 2381 mg (76.9 mmol) phosphate per week must be prevented by other means to prevent phosphate overload.

In reality, the dietary phosphate intake of many dialysis patients is likely to be closer to the typical US daily phosphate intake of 1300 mg (41.9 mmol elemental phosphorus) per day^14^; furthermore, the gastrointestinal tract may absorb up to 86% of ingested phosphate depending on vitamin D status.^13^ Add to this the problem of phosphate-based food additives that are better absorbed than the phosphates found in unprocessed foods,^15^ and the phosphate burden could be significantly higher than the above estimates.

Effective dietary phosphate binders are therefore desirable to prevent phosphate overload.^7^,^16^ In our studies in healthy volunteers, lanthanum (given as LC) reduced urinary phosphorus excretion at all doses tested; 3000 mg/day produced a near-maximal response, with saturation occurring at higher doses. These results are consistent with clinical practice where doses of 2250–3000 mg/day appear to be effective for management of hyperphosphatemia.

The data reported here suggest that the standard 1000 mg dose of LC reduces mean 24-h urinary phosphorus excretion by between 79 and 156 mg/day (2.5 and 5.0 mmol/day) in healthy volunteers (data from Table 4 and Figure 1). In the studies in which they were directly compared, the different LC formulations were shown to be pharmacodynamically equivalent and there were no notable differences between the formulations. There was some inner study variation despite the same LC dose, probably due to differences in study procedures, the race of participants,^17^ geographical location,^18^ the standardized phosphate content of diets, and potentially the type of phosphate (organic vs. inorganic) found in these diets.^19^ The largest reduction in 24-h urinary phosphorus excretion was experienced in the Japanese participants in study 5. A recent study has shown racial differences in the renal handling of phosphate between Caucasian and Black individuals^17^; phosphate handling may also be different in Asian populations. The use of a standardized diet with similar phosphate content to that of the other studies in Japanese individuals with a lower body mass (Table 3) may also have affected phosphate handling. Geographic and seasonal differences in ultraviolet light exposure across study populations may also have resulted in different vitamin D status,^18^ potentially influencing phosphate absorption.

A typical hemodialysis patient may require removal of about 1800 mg (58.1 mmol) of phosphate by binders every week, or approximately 257 mg/day (8.3 mmol/day). Our data suggest that a 1000 mg dose of LC binds 79–156 mg (2.5–5.0 mmol) phosphate; therefore, approximately 1650–3250 mg/day LC would be required to remove a typical phosphate burden. This range correlates reasonably well with clinical experience, as LC doses of 2250–3000 mg/day (typically three tablets per day) appear to be sufficient for management of hyperphosphatemia in the majority of patients.

Several studies have examined the binding capacities of other phosphate binders in healthy volunteers. While comparisons across these studies cannot provide robust evidence, they provide useful indications regarding the relative phosphate-binding capacities of LC and other binders. Burke et al.^5^ administered various doses of SH to healthy volunteers (*n* = 6 per group) receiving a standardized diet (1200 mg elemental phosphorus, similar to most of our studies). Compared with placebo, SH 1000, 2500, and 5000 mg administered three times daily reduced urinary phosphorus excretion by 108 mg/day (3.4 mmol/day), 245 mg/day (7.7 mmol/day), and 340 mg/day (10.6 mmol/day), respectively. Therefore, the apparent phosphate-binding capacity of sevelamer decreased with increasing dose, similar to LC doses greater than 1000 mg three times per day in study 1. A single 1000 mg dose of SH (the most effective dose per gram of binder in the Burke study) was estimated to result in a reduction in mean 24-h urinary phosphorus of approximately 36 mg/day (1.2 mmol/day), equivalent to 29 mg/day (0.9 mmol/day) per 800 mg tablet. No published data on the binding capacity of the alternative sevelamer carbonate formulation compared with SH are available.

Phosphorus recovered in urine over 6 h after a 1000 mg (32.3 mmol) phosphorus load and administration of calcium phosphate binders has been assessed in six healthy volunteers.^20^ Dosing with two calcium carbonate tablets (800 mg of elemental calcium) resulted in a decrease in urinary phosphorus excretion of 31 mg (1.0 mmol) compared with control, whereas six calcium acetate tablets reduced urinary phosphorus by 132 mg (4.3 mmol) [22 mg (0.71 mmol) per tablet]. In another study^21^ the calculated reduction in 24-h urinary excretion of phosphate after 6 days of treatment was 66 mg (2.1 mmol) per 1000 mg of elemental calcium compared with 32 mg (1.0 mmol) per 1000 mg of SH.

Following consumption of a set meal (daily elemental phosphorus intake of 1000 mg in three meals), net phosphorus absorption was determined based on fecal phosphorus excretion.^16^ In this study, 1000 mg of elemental calcium (given as calcium acetate) bound 174 mg (5.6 mmol) of phosphorus [equating to 29 mg (0.9 mmol) per tablet], whereas the same elemental dose of calcium given as the carbonate bound 112 mg (3.6 mmol) of phosphate. Calcium citrate bound 92 mg (3.0 mmol) of phosphorus per 1000 mg of elemental calcium and aluminum carbonate bound 155 mg (5.0 mmol) of phosphorus per 1000 mg of elemental aluminum.

Only indirect comparisons of phosphate-binding capacity can be made based on our studies and the published literature. To bind the 257 mg/day (8.3 mmol/day) excess phosphate from a dietary intake of 1000 mg/day (32.3 mmol/day), a hemodialysis patient may theoretically require approximately three LC (3000 mg elemental lanthanum) tablets, nine calcium acetate tablets (1521 mg elemental calcium), or nine SH tablets (7200 mg SH). These calculations are consistent with manufacturers' dosage recommendations^22^–^24^ and reflect clinical practice where phosphate binders often contribute 50% of total daily tablet burden, which may exceed 25 tablets per day in some patients.^25^

The proposition that urinary phosphorus excretion is an index for phosphate binding assumes that the individual is at steady state. Little is known about the time effects of phosphate binders on net phosphate absorption. Calcium absorption has been better studied; it takes between 10 and 17 days to achieve a new steady state after a change in calcium intake.^26^ Phosphate may achieve steady state more rapidly because the renal mechanism for phosphate conservation is more responsive to phosphatonins and does not require new protein synthesis. Thus, short-term studies serve as an effective estimate of the relative magnitude of phosphate binding by various agents, but unless studies occur within the same individuals under identical conditions, the results should only be used as a first estimate of relative binding capacity. Thus, there are clear limitations when comparing data from different studies and a direct comparison of phosphate binders in healthy volunteers is required. It should be noted, however, that patients with CKD may differ from healthy individuals in terms of gastrointestinal absorption dynamics, such as transit times, effects of pH on binding and absorption, and drug interactions. Relative phosphate-binding capacities in individuals with CKD may therefore differ from those established in studies in healthy volunteers.

In conclusion, LC effectively reduces absorption of dietary phosphate in healthy individuals. Clinicians should consider the dose–response relationship for the different phosphate binders when reviewing doses or choice of binder. A direct comparison of the binding capacities of currently available phosphate binders would be useful to guide clinical practice.
